# Clinical Management of Synthetic-Cannabinoid-Induced Psychosis: A Systematic Review of Treatment Strategies and Outcomes

**DOI:** 10.3390/brainsci15091006

**Published:** 2025-09-17

**Authors:** Alessio Mosca, Stefania Chiappini, Andrea Miuli, Clara Cavallotto, Mauro Pettorruso, Giovanni Martinotti, Fabrizio Schifano

**Affiliations:** 1 Department of Neuroscience, Imaging and Clinical Sciences, “G. d’ Annunzio” University, 66100 Chieti, Italy; alessio.mosca909@gmail.com (A.M.); andreamiuli@live.it (A.M.); mauro.pettorruso@hotmail.it (M.P.); giovanni.martinotti@gmail.com (G.M.); 2 Psychopharmacology, Drug Misuse and Novel Psychoactive Substances Research Unit, School of Life and Medical Sciences, University of Hertfordshire, Hatfield AL10 9EU, UK; stefaniachiappini9@gmail.com (S.C.); f.schifano@herts.ac.uk (F.S.); 3 School of Medicine, UniCamillus International University of Medical Sciences, Via di S. Alessandro 8, 00131 Rome, Italy

**Keywords:** drug abuse, drug misuse, new psychoactive substances, NPSs, synthetic cannabinoids, substance-induced psychosis, Spice, cannabinoid-induced psychosis, treatment, medication

## Abstract

Background: Synthetic cannabinoid receptor agonists (SCRAs, commercially known as “Spice”) have become a leading cause of substance-induced psychosis worldwide. These compounds show strong associations not only with acute psychotic episodes but also, in a subset of patients, with persistent or relapsing psychotic disorders, patterns that raise concern about progression to schizophrenia. Yet clinicians still lack clear, evidence-based guidance, and the optimal management of SCRA-induced psychosis remains inadequately defined. Methods: We carried out a systematic search of PubMed, Scopus, and Web of Science on 2 April 2025, identifying 35 primary studies that together describe roughly 4600 clinical presentations (≈77% male; mean age: 24.7 years). Results: Across diverse settings a convergent three-step pharmacological strategy emerged. First, rapid tranquillization with parenteral benzodiazepines consistently controlled severe agitation and autonomic instability. Second, when florid psychosis persisted beyond 30–60 min, clinicians introduced a second-generation antipsychotic—most commonly olanzapine, risperidone, or aripiprazole—often at doses exceeding those used for primary psychoses. Third, for the minority of refractory or relapse-prone cases, escalation to long-acting injectable formulations or low-dose clozapine achieved symptom control, even at plasma levels below those required in treatment-resistant schizophrenia. Although the evidence base consists largely of uncontrolled clinical descriptions, across studies, a recurrent clinical pattern was observed: initial benzodiazepines for agitation, followed by antipsychotics when psychosis persisted and escalation to clozapine or long-acting injectables in refractory cases. This approach appears to be associated with symptom improvement, although the certainty of the evidence is low to very low. Conclusions. Prospective, comparative studies are urgently needed to refine dosing, directly compare antipsychotic classes, and evaluate emerging cannabinoid-modulating interventions.

## 1. Introduction

Synthetic cannabinoid receptor agonists (SCRAs) have emerged as one of the most troublesome groups of novel psychoactive substances (NPSs) in the last two decades. Marketed under names such as “Spice,” “K2,” or “legal highs,” these laboratory-engineered molecules bind to CB1 receptors with a far greater affinity than Δ^9^-tetrahydrocannabinol (THC), producing potent, and often unpredictable, psychoactive effects [1]. Typically sprayed onto herbal material or dissolved in vaping liquids of unknown concentration, SCRAs are easily purchased online or in street markets, bypassing traditional drug-control statutes and routine toxicology screens [2].

Clinically, SCRA intoxication has become synonymous with severe, rapidly evolving psychosis [1]. Emergency departments, critical-care units, military and prison clinics, and psychiatric wards worldwide now report acute presentations characterized by delusion, extreme agitation, aggression, catatonia, or dissociation [1]. In vulnerable users, a single exposure can precipitate de novo substance-induced psychotic disorder; repeated use is linked to relapses of primary psychotic illnesses and persistent substance-related exogenous psychosis [3,4,5]. Beyond the psychiatric sequelae, SCRAs are associated with life-threatening medical complications—including seizures, hyperthermia, acute kidney injury, myocardial infarction, and stroke—further complicating clinical management [6].

While SCRAs belong to the broader constellation of NPSs, a phenomenon that has been constantly rising since 2000 [7] and now includes more than 1200 substances documented by the European Union Drugs Agency (EUDA), they account for a disproportionate share of emergency toxicology alerts.

The United Nations Office on Drugs and Crime (UNODC) and the European Union Drugs Agency (EUDA) define NPSs as “substances of abuse, either in pure form or in preparations, that are not controlled by the 1961 Single Convention on Narcotic Drugs or the 1971 Convention on Psychotropic Substances but may pose a public health threat”. These substances are often synthesized to mimic the psychoactive effects of controlled substances such as cannabis, cocaine, ecstasy, and lysergic acid diethylamide (LSD), bypassing existing drug regulations (they are marketed as “legal highs”) and making their detection at routine screening tests and control more challenging [2]. In addition to SCRAs, the spectrum of NPSs encompasses a diverse array of compounds, including synthetic cathinones; phencyclidine-like arylcyclohexylamines; phenethylamines; piperazines; tryptamines; aminoindanes; various novel opioids and benzodiazepines; and dissociatives such as benzydamine, which have been increasingly misused for their hallucinogenic properties [8]. Benzydamine, in particular, has emerged as a substance of abuse, especially among adolescents, due to its low cost, accessibility, and dissociative effects at high doses [9].

Although frequently promoted as “legal” replacements for conventional drugs of abuse, these molecules are characterized by scarce safety data, highly variable and often severe acute toxic effects, and substantial potential for psychiatric complications.

Online “psychonaut” forums and encrypted marketplaces accelerate the global diffusion of each new analog, ensuring that local clinicians often face unfamiliar molecules with scant pharmacological data [10,11]. One of the principal challenges in managing intoxications caused by novel psychoactive substances (NPSs) is the marked discrepancy between the severity of the clinical presentation and the lack of a corresponding analytical confirmation [12]. Routine toxicology screens seldom detect designer benzodiazepines or the newest synthetic opioids [13,14]; as a result, clinicians are often unable to decide with confidence whether to administer targeted antagonists such as flumazenil or naloxone, or to gauge the need for additional pharmacologic interventions [15]. Empirical drug administration, moreover, can interact unpredictably with ingested NPSs, exposing patients to adverse cardiovascular events (e.g., arrhythmias), neurological complications (e.g., seizures), or neurotransmitter-excess syndromes such as serotonin syndrome [16].

With respect to SCRA consumption, one of the most critical safety issues is their pronounced psychiatric toxicity. Reported outcomes include the emergence of acute psychotic syndromes, self-injury, suicidal behavior, dependence with ensuing withdrawal phenomena, and even life-threatening intoxication or overdose [8,17]. Particularly disquieting is the appearance of de novo psychosis in susceptible individuals, defined as substance/medication-induced psychotic disorder (SIPD) [3,17], and the capacity for these agents to precipitate schizophrenia or other primary psychotic disorders (PPDs) [4]. In addition, mounting evidence points to the persistence of substance-related exogenous psychoses (SREPs) that may outlast the period of acute use [4,6]. Dissociation is another typical symptom. It is more pronounced and clinically relevant than that observed with classical cannabis [7] and is influenced by premorbid psychiatric conditions, as evidenced with cannabis [18].

Emerging therapies are being investigated for their potential applicability in this population. Notably, studies on ibogaine and its metabolite noribogaine conducted in individuals with substance use disorders—including those with SCRA and polysubstance abuse—have shown promise in reducing cravings, impulsivity, and psychiatric symptomatology [19]. Similarly, lurasidone and brexpiprazole have been evaluated in schizophrenia-spectrum patients with co-occurring alcohol or substance use disorders, demonstrating significant clinical and functional benefits that may support their off-label consideration in SCRA-related psychotic crises [20,21]. However, more data are needed to clarify their role in the acute setting of NPS intoxication.

Despite the mounting caseload, evidence-based guidance for front-line clinicians remains strikingly sparse. Decisions about rapid tranquillization, antipsychotic selection and dosing, adjunctive benzodiazepines, intensive monitoring, and post-discharge care are largely extrapolated from anecdotal reports or small case series, leading to heterogeneous practices and uncertain outcomes.

*Aim of this study*: The present systematic review therefore catalogues and critically appraises all interventions reported for the management of SCRA-induced psychosis. Specifically, we examine the therapeutic strategies reported in the literature, including both acute interventions and longer-term management plans, and evaluate their effectiveness and safety profiles. By qualitatively analyzing treatment approaches across published cases and studies, we seek to identify best-practice patterns and highlight any pharmacological or psychosocial measures that appear especially useful for mitigating psychotic symptoms provoked by synthetic cannabinoid use.

## 2. Materials and Methods

### 2.1. Systematic Review Procedures

A systematic electronic search was performed on 2 April 2025 on the following search engines: PubMed, Scopus, and Web of Science (WoS). Other relevant papers not resulting from the described search were added from the references of the included articles. For PubMed and WoS the following search strategy was used: [(“synthetic cannabinoids” OR “spice”) AND (“psychosis” OR “hallucination” OR “delusion” OR “schizophrenia” OR “delusional” OR “schizoaffective”) NOT review NOT animal]. For Scopus a slightly different search strategy was used: TITLE-ABS-KEY (“synthetic cannabinoids” OR “spice”) AND TITLE-ABS-KEY (“psychosis” OR “hallucination” OR “delusion” OR “schizophrenia” OR “delusional” OR “schizoaffective”) AND NOT TITLE-ABS-KEY (review) AND BOT TITLE-ABS-KEY (animal). No date restrictions were applied, and all available years were considered. Only studies published in English were included.

The systematic review was structured in accordance with the PRISMA guidelines [22]. The identified studies were assessed based on their titles/abstracts and full-text screening against eligibility criteria.

Only original articles written in English that report data on treatment and management strategies for synthetic-cannabinoid-induced psychosis were included. By collating and critically appraising the available literature, this review aims to (i) map the range of pharmacological and nonpharmacological interventions reported, (ii) evaluate their apparent clinical outcomes and adverse-effect profiles, and (iii) identify gaps to inform future research and guideline development.

### 2.2. Protocol and Registration

The current research methods were registered in PROSPERO (identification code: CRD420251107913. 

### 2.3. Eligibility Criteria (PICO Framework)

Population (P): Humans presenting with psychosis temporally associated with exposure to synthetic cannabinoids (SCRAs).

Intervention (I): Any clinical management strategies, including pharmacological treatments (benzodiazepines, antipsychotics, clozapine, long-acting injectables, and other agents) and nonpharmacological interventions (supportive care, psychoeducation, counseling, and referral to addiction services).

Comparison (C): None, standard care, or other treatments when available.

Outcomes (O): Resolution of acute psychosis, persistence or relapse, adverse events (including ICU admission or death), and longer-term functional outcomes when reported.

### 2.4. Data Synthesis Strategy

The selection and eligibility phase of the protocol was carried out independently by A.M. (Alessio Mosca), A.M. (Andrea Miuli), and C.C. after a final cross-check by S.C. and M.P. All discordant cases were evaluated by G.M and F.S. Any remaining doubts related to the topics covered in the articles were clarified directly by the authors, if contactable. Data were extracted into structured Word tables using a predefined set of variables: first author and year of publication, study design, patient demographics (age and gender), details of SCRA exposure (substance, dose, and route of administration), presence of psychiatric comorbidities and concomitant substance use, clinical presentation and psychiatric symptoms, treatments administered, outcomes (acute remission, persistence, and relapse), follow-up duration, and authors’ recommendations for clinicians.

**Figure 1:**
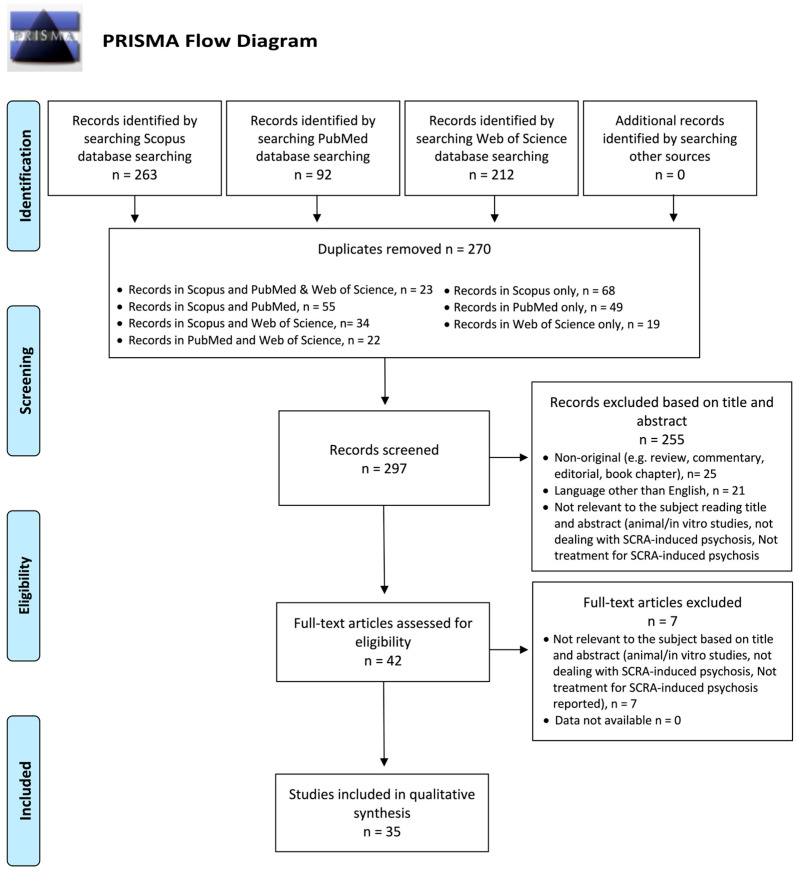
A PRISMA flow diagram of the methodology of the systematic literature review.

The exclusion criteria for both selection phases were (1) non-original research (e.g., reviews, metanalyses, commentaries, editorials, letters to the editor without data available, and book chapters); (2) non-full-text articles (e.g., meeting abstracts); (3) languages other than English; (4) animal/in vitro studies; (5) articles not dealing with SCRA-induced psychosis; and (6) no treatment for SCRA-induced psychosis reported. From a total of 231 articles (PubMed = 92; Scopus = 263; WoS = 212; other sources = 0), after deduplication (n = 52), 297 records were screened. Among the articles screened, 255 were not considered relevant to the subject based on the titles and abstracts. Of the 42 full-text articles assessed for eligibility, 5 did not match the inclusion criteria for our review and 2 were not available (Appendix A). Finally, 35 articles were included in the systematic review (Figure 1).

### 2.5. Risk of Bias and Quality of Evidence

Risk of bias was assessed according to the study design. For randomized trials we planned to use RoB 2 [23]; for non-randomized observational studies we applied ROBINS-I [24], covering seven domains (confounding, selection of participants, classification of interventions, deviations from intended interventions, missing data, measurement of outcomes, and selection of the reported result). For case reports and case series, we used the CARE checklist [25], which evaluates the completeness of a clinical description (including the title, abstract, timeline, follow-up, patient perspective, and informed consent).

## 3. Results

### 3.1. General Features

A total of 35 studies were included in the present systematic review [26,27,28,29,30,31,32,33,34,35,36,37,38,39,40,41,42,43,44,45,46,47,48,49,50,51,52,53,54,55,56,57,58,59,60]. Findings related to the 35 articles are described in detail and organized based on the specific molecules and the alphabetical order of the authors (Table 1). To improve table readability and highlight the treatment focus, while preserving the completeness of the results, a table identifying the specific SCRAs involved was placed in Appendix B. Correspondingly, psychiatric comorbidity is detailed in Appendix C, and patterns of polydrug use are presented in Appendix D.

Among the 35 primary reports, 18 were single-patient case reports, 11 were case series (2–16 patients), 5 were observational cohorts/cross-sectional audits (17–1.898 presentations), and 1 was a small open-label intervention study. The combined sample comprised ≈ 4600 individuals; males predominated (≈77%), and the weighted mean age was 24.7 years (range: 14–70). Smoking was the route of administration in 94% of the publications; two studies also described oral or intranasal use.

### 3.2. Risk of Bias

The non-randomized observational studies generally showed a serious risk of bias, mainly due to uncontrolled confounding (e.g., polysubstance use and psychiatric comorbidity), patient selection issues, and heterogeneous outcome measurement. The case reports and case series were of variable quality: while most provided adequate clinical details, timelines of events and the patient perspective were frequently missing (see Appendix E (ROBINS-I Assessment) and Appendix F (CARE Checklist—Case Reports)). These limitations substantially lower the overall certainty of the evidence, as further discussed below.

### 3.3. Clinical Presentation

Across all designs, the index presentation was an acute psychotic syndrome with severe psychomotor agitation, often accompanied by anxiety, suicidality, or catatonic features. Agitation/aggression was explicitly mentioned in 31/35 papers, while persecutory or grandiose delusions were mentioned in 27/35, and complex visual hallucinations were mentioned in 17/35. Disturbances in thought processes—such as paranoia, thought blocking, and delusional thinking—were frequently documented. Some reports also described complex delusional states (e.g., Capgras syndrome and mystical or supernatural delusions), negative symptoms (e.g., flat affect, alogia, and avolition), and dissociative phenomena. Autonomic instability (tachy-/bradycardia, hypertension, and seizures) and electrolyte disturbances drove admission to intensive care in 5–25% of the emergency-department cohorts.

### 3.4. Pharmacological Management

Pharmacological management of Spice-induced psychosis follows a pragmatic, stepped-care model. Initial control of agitation and autonomic instability is almost universally achieved with parenteral benzodiazepines, which, as sole agents, are sufficient for mild intoxications that remit within a few hours. When frank psychosis persists beyond the immediate sedation window, clinicians typically introduce an antipsychotic—nowadays favoring second-generation agents such as olanzapine, risperidone, or aripiprazole—at doses higher than those used for cannabis-related or primary psychoses. This combination allows faster resolution and reduces the need for prolonged restraint. In the minority of cases that prove refractory or are complicated by poor adherence, escalation to long-acting injectable formulations or low-dose clozapine has shown reliable efficacy, often at lower plasma exposures than required for treatment-resistant schizophrenia. Across more than 4500 documented presentations, this tiered approach yielded rapid symptom clearance in approximately 90% of patients within four days, with serious adverse events and intensive care unit-level complications remaining rare; when relapse occurred, it was almost invariably linked to renewed Spice consumption rather than treatment failure. Collectively, the evidence—though derived mainly from case reports and observational cohorts—supports a simple algorithm: prompt benzodiazepine sedation, early antipsychotic augmentation for ongoing psychosis, judicious use of clozapine or depot preparations for resistant or recurrent episodes, and rigorous counselling aimed at sustained abstinence. These findings are presented in Table 2.

### 3.5. Treatment Sequencing

**Figure 2:**
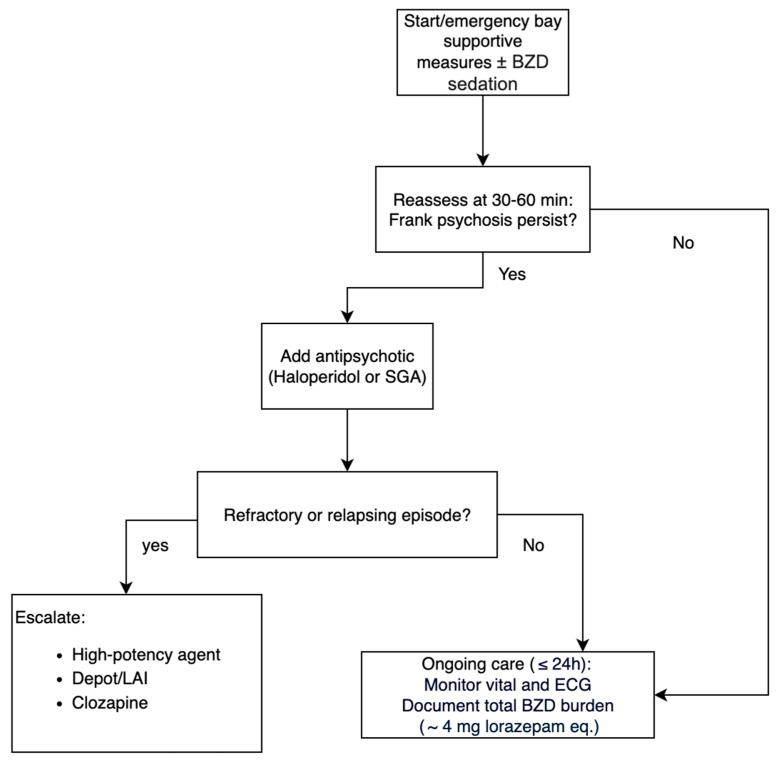
Acute management.

All studies converged on a stepwise algorithm: (1) supportive measures ± BZD sedation; (2) add an antipsychotic if frank psychosis persists for >30–60 min; and (3) escalate to high-potency or depot formulations, or clozapine for refractory or relapsing cases. Haloperidol or SGAs were usually introduced during the first day of hospitalization. The median total benzodiazepine burden before antipsychotic introduction was 4 mg of lorazepam equivalents (see Figure 2).

### 3.6. Outcome and Course

Acute symptoms resolved in 90% of cases within 24–96 h. Twelve reports (≈180 patients) documented persistent or relapsing psychosis lasting weeks to months, which was invariably linked to continued Spice use or a pre-existing psychotic disorder. Relapse occurred in 100% of people who resumed consumption. The antipsychotic that was effective during the index episode was successful again on readmission. ICU-level complications (status seizures, rhabdomyolysis, and acute kidney injury) were uncommon (<5%) and largely confined to polysubstance users. There were no treatment-attributable deaths; one observational cohort recorded a single fatality due to multi-organ failure before any psychotropic was administered.

### 3.7. Adverse Events and Safety

Across the included studies, the most commonly reported adverse effects during psychopharmacological management were sedation, extrapyramidal symptoms (particularly with first-generation antipsychotics), and QT prolongation requiring ECG monitoring. Benzodiazepines were generally well tolerated, with rare reports of respiratory depression requiring close observation. Second-generation antipsychotics (e.g., risperidone, olanzapine, and quetiapine) were usually safe, although isolated cases of neuroleptic malignant syndrome and cardiovascular effects were reported. Clozapine use necessitated monitoring for hematological adverse effects, though no clozapine-related agranulocytosis was documented.

A minority of the patients required ICU-level interventions, mainly due to status seizures, rhabdomyolysis, or acute kidney injury. Importantly, no treatment-attributable deaths were reported in the included literature.

## 4. Discussion

This systematic review collates and critically appraises the entire body of evidence related to managing psychosis precipitated by SCRAs. These compounds are high-potency full cannabinoid (CB_1_) receptor agonists that lack cannabidiol’s buffering effect, creating a neurochemical milieu that strongly favors psychosis [4,55,61], a pattern also reported with other NPSs and emerging drugs of abuse [19].

Our review confirms that SCRAs can elicit a broad spectrum of psychopathology, ranging from mild perceptual changes and affective disturbances to acute psychotic episodes clinically indistinguishable from primary psychotic disorders, including schizophrenia. A marked sex imbalance was evident, with most cases involving men, consistent with earlier observations [62]. Nevertheless, the age range was wide—from adolescents as young as 14 years [28] to adults aged 70 years [60]—highlighting the need to consider SCRA use as a psychosis risk factor across the lifespan.

Despite heterogeneous, largely descriptive data, a consistent three-step pharmacological pathway emerged. First, almost all reports used parenteral benzodiazepines to control severe agitation and autonomic instability [44,54]. Second, if florid psychosis persisted beyond 30–60 min, the clinicians introduced a second-generation antipsychotic—most often olanzapine, risperidone, or aripiprazole—typically at higher doses than those used for primary psychoses [33,56]. Third, a minority of the refractory or relapse-prone cases required escalation to long-acting injectables or low-dose clozapine [51,52]. This potential intervention should be carefully monitored, given the risk of cardiac complications associated with clozapine use [63] in a population already at risk of cardiorespiratory issues. This algorithm achieved full or near-full remission within 24–96 h in ≈90% of the ≈4600 documented presentations, with serious drug-related adverse events remaining uncommon (<5%) [42,47].

These findings are consistent with previous research advocating for acute use of benzodiazepines to control psychotic agitation [64,65]; employment of atypical antipsychotics in dual-diagnosis populations [66], alone or in combination with benzodiazepines [67]; and preferential use of clozapine for the most severe forms of early psychosis [68].

Despite these findings providing useful indications for clinical practice, the overall certainty of the available evidence remains low to very low. This is mainly due to the serious risk of bias identified in the non-randomized studies (confounding, patient selection, and heterogeneous outcome measurement) and the incomplete reporting in many of the case reports (such as the frequent absence of timelines and patient perspectives noted with the CARE checklist). Therefore, although consistent clinical patterns can be observed, they should be interpreted with caution and regarded as hypothesis-generating rather than confirmatory.

Routine screening for NPSs, including SCRAs, should be standard in first-episode or atypical psychosis assessments, and toxicology laboratories need assays that keep pace with the rapidly evolving roster of SCRA analogs [47]. At the population level, stronger regulation, harm-reduction campaigns, and targeted education are essential to mitigate the psychiatric burden associated with these compounds.

Beyond established pharmacotherapy, some authors have proposed cannabinoid-modulating “antidotes,” such as the CB_1_ antagonist rimonabant or cannabidiol, for acute SCRA intoxication, including psychosis, but evidence is preliminary and potential psychiatric risks warrant caution [69]. These agents should therefore be regarded as experimental until evaluated in well-designed comparative studies.

Several reports described successful management of acute SCRA-induced psychosis with supportive care alone, including a low-stimulus environment, verbal de-escalation, hydration, and close observation, without the need for psychotropic medication. This highlights the importance of considering nonpharmacological strategies, particularly in emergency settings where agitation and autonomic instability may resolve spontaneously. In addition, long-term management should include psychoeducation, abstinence-oriented counselling, and referral to addiction services, which are essential to reduce relapse risk and improve global functioning, consistent with evidence from broader psychosis populations showing the benefits of psychosocial and psychological interventions when combined with pharmacotherapy [70,71].

Persistent or relapsing psychotic states, sometimes termed “Spiceophrenia”, have been documented [4,6]. These outcomes appear to be linked less to treatment quality than to individual vulnerability to develop chronic psychosis. Long-term studies involving other substance-induced psychoses indicate that 25–66% of cases eventually convert to a primary psychotic disorder [72,73,74,75], underscoring the need for structured follow-up.

### Limitations

As mentioned in the discussion, the overall certainty of the available evidence is low to very low. This is primarily due to the serious risk of bias identified across the non-randomized studies and the incomplete reporting in many of the case reports. Another important limitation of the current evidence is the short follow-ups reported in most of the studies. In the majority of the case reports and observational series, follow-up was restricted to the acute hospital stay or a few weeks after discharge. Another limitation concerns the numerical synthesis: large cohorts [29,43,47] dominate the overall totals, and in at least one study [39] the unit reported was admissions rather than individual patients, potentially inflating the numbers. Likewise, averages for age and gender were derived from heterogeneous sources and should be interpreted with caution.

Another limitation is that some elements of the proposed management algorithm are extrapolated from the broader psychosis and agitation literature, rather than being directly supported by SCRA-specific studies. This distinction is essential to avoid overinterpreting the evidence and to clarify where clinical extrapolation begins. This prevents any firm conclusions about the long-term prognosis of SCRA-induced psychosis, including relapse risk, chronic trajectories, and functional outcomes. The proposed algorithm should be regarded as a hypothesis-generating synthesis derived from heterogeneous and predominantly low-quality evidence. Its apparent consistency across the case reports and small observational studies is informative, but validation in prospective, comparative studies is absolutely necessary before any clinical recommendations can be made. Future prospective studies with extended follow-up periods will be essential to address this gap. A further limitation is the frequent presence of polysubstance use and psychiatric comorbidities among the reported cases. These factors make it difficult to attribute symptoms and outcomes exclusively to SCRA exposure. However, as they reflect real-world clinical scenarios, we chose to include such studies while documenting polysubstance use (Appendix D) and psychiatric comorbidities (Appendix C) separately. Their potential confounding role is acknowledged in our interpretation of the outcomes.

## 5. Conclusions

This synthesis is essential to support emergency physicians, psychiatrists, and addiction specialists, who increasingly encounter SCRA-related psychosis, and to enhance patient safety in this evolving area of substance-induced mental health care. Prospective, comparative trials are urgently needed to refine the optimal dosing; directly compare first- and second-generation antipsychotics; and clarify the therapeutic potential of CB_1_ antagonists, CBD, and other neuromodulators. Longitudinal cohort studies should characterize neurobiological substrates, genetic vulnerability, and trajectories from acute SCRA-induced psychosis to enduring psychotic disorders. Such research will be indispensable for developing robust, evidence-based guidelines and ultimately improving outcomes in this rapidly evolving area of toxicology and psychiatry.

## Figures and Tables

**Table 1:** Main findings.

Name, Year	Study Design	Population (N, M, F)	Mean Age ± Standard Deviation	Administration	Psychiatric Symptoms	Treatment	Outcome	Recommendation for Clinicians
**Abouchedid et al., 2016 [26]**	Case report	F = 1	19	Smoked	Unspecified visual hallucinations	Single dose of midazolam (1 mg IV) stopped seizures/agitation; no further medication needed.	Acute psychosis with remission	A low dose of IV benzodiazepine is often sufficient for SCRA-induced convulsions or severe agitation.
**Altintas et al., 2016 [27]**	Single-center cross-sectional analysis	M = 50	MA = 25.9 ± 5.5	NA	Suicidal ideation, suicide attempt	Standard antipsychotic treatment in an acute psychiatric ward (agents not specified)	Acute psychosis with remission	Manage SCRA psychosis as primary psychosis but expect an earlier age of onset and monitor suicidality closely.
**Barceló et al., 2017 [28]**	Case series	N = 5 M = 4 F = 1	**Case 1** = 17 **Case 2** = 17 **Case 3** = 17 **Case 4** = 14 **Case 5** = 21	Smoked	Agitation, confusion, anxiety, suicide attempt, altered language, bradypsychia, delusions of influence and grandeur	Three 17-year-old boys: Verbal reassurance/observation only—no drugs required 14-year-old girl and 21-year-old man: Intravenous crystalloid fluids	Acute psychosis with remission	Most mild-to-moderate SCRA intoxications settle within hours; start with calm environment and IVFs for dehydration/tachycardia. Admit only if neuro-psychiatric symptoms persist or airway risk develops.
**Bassir et al., 2016 [29]**	Retrospective review	N = 594 M = 444 F = 150	MA = 40.6 ± 12.9	Smoked	Agitation, suicidal ideation, mood symptoms, thought disorganization, internal preoccupation	SC-only patients required higher antipsychotic doses and longer psychiatric admissions than cannabis users. Exact drugs not specified.	NA	In SCRA users with severe psychosis, start antipsychotics at the upper end of the dosing range. Plan follow-up for sustained abstinence and psychosocial support.
**Bebarta et al., 2012 [30]**	Case series	**Case 1** = M **Case 2** = 2 **Case 3** = M	**Case 1** = 19 **Case 2** = 19 **Case 3** = 23	Smoked	Aggression, agitation, panic, sedation, paranoia, visual and somatic hallucinations	Three service members: IV lorazepam (2 mg) for severe agitation (Case 1); naloxone trial in sedated patient (Case 2, no effect); IV fluids, oxygen, overnight ward observation for all.	Acute psychosis with remission	Provide airway support, hydrate generously, and give benzodiazepines for agitation.
**Berry-Cabàn et al., 2013 [31]**	Case report	M = 1	20	Smoked	Thought blocking, disorganized thinking and behaviors, paranoid delusion, referential delusion, loss of ego boundaries, verbal hallucinations	ED/ward: Repeated lorazepam (≥5 mg total) for severe agitation + restraints; diphenhydramine (25 mg) + haloperidol (5 mg) IM. Later, risperidone (1 mg/night) for residual psychosis.	Onset with persistence	Manage SCRA-induced delusions with rapid benzodiazepine sedation first. Add parenteral antipsychotic if psychosis persists. Anticipate prolonged cognitive blunting and arrange close supervision.
**Besli et al., 2015 [32]**	Case series	N = 16 M = 15 F = 1	MA = 15.4 ± 1.7	Smoked	Agitation, anxiety, panic attack, numbness, euphoria, sympathomimetic symptoms, perceptual changes	Pediatric ED management (*n* = 16): IV crystalloids, benzodiazepines PRN for agitation. In total, 25% required ICU monitoring for hypotension, brady-/tachycardia. Social-work referrals for all.	Acute psychosis with remission	Treat adolescent SCRA intoxication like any unknown toxidrome: stabilize airway/BP, give benzodiazepines for neuro-behavioral control, and admit to ICU if vitals are labile. Education and early addiction follow-up are critical.
**Bonaccorso et al., 2018 [33]**	Case series	**Case 1** = M **Case 2** = F **Case 3** = M **Case 4** = M	**Case 1** = 28 **Case 2** = 32 **Case 3** = 20 **Case 4** = 39	Smoked	Agitation, verbal and physical aggression, sexual disinhibition, disorganization, bizarre behavior, delusional mood, persecutory and grandiose delusions, auditory hallucinations	Combination regimens: Olanzapine (up to 20 mg/day), aripiprazole (9.75 mg tds), haloperidol (10 mg/day), depot zuclopenthixol (300 mg/week), clonazepam (≤8 mg/day), lithium (800 mg), sodium valproate (1200 mg).	Acute psychosis with remission/psychotic relapse	Administer first-line BDZ for agitation, then add high-dose SGA (avoid QT-prolonging FGA where possible). Monitor vitals with NEWS ≥ TDS, and tighten observation/leave until urine screens are negative.
**Celofiga et al., 2014 [34]**	Case series	M = 4	**Case 1** = 35 **Case 2** = 21 **Case 3** = 27 **Case 4** = 29	Smoked	Agitation, mood changes, anxiety, elevated affect, chronic paranoid and grandiose delusions, bizarre behavior, formal thought symptoms, haptic hallucinations	Escalation of existing benzodiazepines: Diazepam (up to 10 mg TID); oral lorazepam (up to 2.5 mg TID or 2 mg IM) for agitation/anxiety. Continued baseline antipsychotics (haloperidol decanoate, risperidone LAI, clozapine, quetiapine, olanzapine)	Acute psychosis with remission/psychotic relapse	In stable patients with psychotic disorders, acute SCRA intoxication is usually managed by temporarily increasing benzodiazepines while maintaining the standing antipsychotic.
**Di Petta et al., 2016 [35]**	Case report	M = 1	28	Smoked	Agitation, suicide attempts, irrational behavior, magical delusions, mystical ideas, bizarre delusions of greatness and persecution, Capgras syndrome, Ekbom syndrome, twilight state of consciousness, visual or auditory hallucinations, illusions	Paliperidone palmitate LAI (150 mg monthly) plus phenomenological psychotherapy	Acute psychosis with remission/onset with persistence	In chronic SCRA users with persistent delusional disorder, a long-acting injectable antipsychotic can stabilize psychosis and improve adherence. Combine this treatment with structured psychotherapy for partial functional recovery.
**Durand et al., 2015 [36]**	Case report	M = 1	23	NA	Agitation, persecutory/mystical delusions	Aggressive IV saline + IV lorazepam (2 mg q6 h after admission) to protect kidneys and calm agitation IM chlorpromazine Haloperidol (30 mg/day) + valproate (1.5 g/day) + lorazepam (6 mg/day) for psychosis	Acute psychosis with remission	Haloperidol (or another potent antipsychotic) plus benzodiazepines can safely control prolonged psychosis/agitation.
**Every-Palmer et al., 2011 [37]**	Cohort study	M = 15	MA = 34 ± 7.9	Smoked	Agitation, disorganization, paranoia, an impulse to do evil things, a sense of the end of the world	No acute drugs given in study; all 15 forensic inpatients were already on maintenance antipsychotics.	Acute psychosis with remission/psychotic relapse	
**El Zahran et al., 2019 [38]**	Case report	M = 1	29	Smoked	Agitation, visual hallucinations	Lorazepam for agitation	Acute psychosis with remission	Give supportive care and a benzodiazepine for behavioral control.
**Glue et al., 2013 [39]**	Retrospective observational study	N = 17 M = 10 F = 7	MA = 26.1 ± 10	NA	Homicidal ideation, affective changes (anxious, depressive), intense suicidal thinking/behavior, paranoia, thought disorder, disorganized behavior	Seventeen admissions (13% of total): Supportive care; those with psychosis received antipsychotics (typical or atypical) and sometimes received antidepressants.	Acute psychosis with remission/psychotic relapse	Start antipsychotics promptly, monitor suicidality, and arrange community follow-up once abstinent.
**Haro et al., 2014 [40]**	Letter to editor/case report	F = 1	19	NA	Laughter forfeit, derealization, depersonalization, movement disorder similar to catatonia, soliloquy with personal hygiene deterioration, self-references, visual hallucinations	Aripiprazole (15 mg/day) + lorazepam + biperiden after drug cessation	Acute psychosis with partial remission	If SCRA use is suspected in first-episode psychosis, start atypical antipsychotic plus high-dose benzodiazepine, add anticholinergic if extrapyramidal/catatonic features appear, and insist on sustained abstinence with psychoeducation.
**Helge Müller et al., 2009 [41]**	Case report	M = 1	25	Smoked	Increased anxiety, delusions of influence		Psychotic relapse	
**Hermanns-Clausen et al., 2017 [42]**	Prospective observational study	N = 44 M = 39 F = 5	MA = 20.5	Oral, smoked, sniffed	Restlessness/agitation, amnesia, anxiety, acute psychosis, self-mutilating behavior	Benzodiazepines for agitation/seizures (17/44 cases) Thiopental ± propofol to terminate refractory convulsions (1 case)	Acute psychosis with remission	Treat SCRA intoxication like a toxic delusion: give IV benzodiazepines early and be ready to intubate or deeply sedate for status seizures.
**Hoyte et al., 2012 [43]**	Observational study	N = 1898 M = 1005 F = 893	MA = 22.5 ± 8.86	Smoked	Agitation, irritability, drowsiness, lethargy, confusion, dizziness, paranoia, unspecified delusions and hallucinations	IV crystalloids ≈ 25%. Benzodiazepines ≈ 16% (for agitation/seizures). In total, >70% required no drug therapy.	Acute psychosis with remission	Most presentations resolve with supportive ED care alone. Use benzodiazepine if the patient is agitated or seizing.
**Hurst et al., 2011 [44]**	Case series	M = 10	MA= 23	Smoked	Insomnia, psychomotor agitation, suicidal ideation, anxiety, flat affect, alogia, paranoid delusions, thought blocking, disorganized speeches and behavior, psychomotor retardation, auditory and visual hallucinations	Antipsychotics given to 7/10 patients (agents not specified; used for active psychosis).	Acute psychosis with remission/onset with persistence	Initiate standard antipsychotic treatment, and monitor because symptoms may persist for weeks or months after intoxication.
**Kekelidze et al., 2019 [45]**	Interventional study	N = 43 M = 38 F = 5	MA = 25	NA	Anxiety; disorientation; dream-like clouding of consciousness; catatonic disorders; catalepsy; profound impairments to consciousness; perceptual delusions; delusional experiences; disorganization; degraded self-awareness; multiple vivid and dynamic pareidolias; visual, tactile, and auditory hallucinations; daydream-like fantastic hallucination	Standard detoxification (IV fluids + B vitamins + nootropics) for all, plus one of the following: Haloperidol (1.5–20 mg/day) (butyrophenone subgroup); Tiapride (100–800 mg/day) (substituted benzamide subgroup); Phenazepam (4–6 mg/day) or diazepam (up to 80 mg/day) for arousal.	Acute psychosis with remission	Choose a neuroleptic by matching it with the psychosis type and the severity of the somato-neurological signs: haloperidol shortens the psychotic phase fastest, whereas tiapride gives quicker relief of autonomic/neurological complications. Always embed antipsychotics in an early, structured detoxification regime.
**Malik et al., 2021 [46]**	Case series	**Case 1** = M **Case 2** = F	**Case 1** = 31 **Case 2** = 36	NA	Aggressivity, bizarre behavior, a delusional self-inflicted injury to the eye	Propofol bolus/infusion to achieve deep sedation for emergency globe-repair surgery. Antipsychotic pharmacotherapy initiated post-operatively (drug not specified).	Acute psychosis with remission/psychotic relapse	In agitated SCRA-induced psychosis with self-harm, use rapid-onset IV anesthetics (propofol or ketamine) to permit life- or organ-saving procedures. Then, transfer to psychiatry for titration of antipsychotics and suicide-risk management.
**Monte et al., 2017 [47]**	Cohort study	N = 353 M = 297 F = 56	MA = 25	NA	Agitation, unspecified delusion	First-line benzodiazepines used in 37% of cases. Antipsychotics used in 10% of cases. In total, 24% of cases required ICU care, with a single recorded fatality.	Acute psychosis with remission	Begin with benzodiazepines for agitation, seizures, or delusion. Add antipsychotics if psychosis persists.
**Oluwabusi et al., 2012 [48]**	Case series	M = 2	**Case 1** = 16 **Case 2** = 17	Smoked	Insomnia, low mood, hyperactivity, anxiety, apathy, paranoid delusions, grandiose delusions, somatic preoccupation, disorganized behavior, auditory and visual hallucinations	Case 1: Initial quetiapine, switched to aripiprazole (20 mg/day); relapse managed with olanzapine ODT titrated to 15 mg/day (symptoms cleared in 72 h). Case 2: Olanzapine (15 mg nightly); recurrence after non-adherence, which resolved again within days after restarting.	Acute psychosis with remission/psychotic relapse	In adolescents with first-episode psychosis linked to SCRAs, start an atypical antipsychotic (olanzapine or aripiprazole) and stress adherence. Screen for ongoing SCRA use and family vulnerability. Early medication plus abstinence usually restores the baseline within days.
**Ozer et al., 2016 [49]**	Case report	M = 1	17	Smoked	Anxiety, agitation, irritability, confusion, insomnia, anorexia, dysphoric mood, suicidality with self-injury, Capgras syndrome, persecutory delusions	Olanzapine (10 mg/day); complete remission within 2 weeks.	Acute psychosis with remission	Atypical antipsychotics (e.g., olanzapine) are effective for SCRA-induced misidentification syndromes.
**Peglow et al., 2012 [50]**	Case report	M = 1	59	Smoked	Traumatic flashbacks; disorganized, bizarre behavior; auditory and visual hallucinations	Observation only, continuing the patient’s usual outpatient regimen (aripiprazole (10 mg), gabapentin, etc.). No additional antipsychotics were required, and the symptoms cleared within 24 h each time.	Acute psychosis with remission	Rule out other drugs, and observe closely. Symptoms may remit rapidly once SCRA use stops.
**Rahmani et al., 2013 [51]**	Case series	M = 2	**Case 1** = 17 **Case 2** = 17	Smoked	Insomnia, irritability, mild agitation, delusion of influence and possession, mystical delusions, a sense of the end of the world, Capgras syndrome, bizarre and disorganized behaviors, auditory and visual hallucinations	Cases 1 and 2: Trials of risperidone, haloperidol, chlorpromazine, valproate, high-dose benzodiazepines (lorazepam/clonazepam) all failed. Switch to clozapine (50–150 mg/day) led to robust improvement.	Acute psychosis with remission/onset with persistence	If SCRAs precipitate a prolonged, antipsychotic-resistant psychosis, consider low-dose clozapine earlier than usual. A therapeutic response may occur at lower doses than in primary schizophrenia.
**Roberto et al., 2016 [52]**	Case report	M = 1	18	Smoked	Confusion, amnesia, agitation, insomnia, catatonia, elevated mood, mutism, avolition, thought disorganization, paranoid delusions, persecution ideation, auditory hallucinations	Lorazepam (2 mg) during first week for catatonia/stiffness. Risperidone orally, titrated to 5 mg/day for acute psychosis, then tapered to 4 mg/day for maintenance. Benztropine (0.5 mg HS) for mild EPSs. Readmission relapse treated with the same risperidone schedule after SCRA abstinence.	Onset with persistence	Start a benzodiazepine promptly when catatonic features are present. Then, introduce a second-generation antipsychotic (e.g., risperidone) and monitor EPSs. The antipsychotic that worked during the index episode will usually work again after relapse if the patient resumes using SCRAs.
**Satodiya et al., 2020 [53]**	Case report	M = 1	32	Smoked	Monotone speech, minimal gestures, social withdrawal, lack of spontaneity, blunted affect, avolition	Optimization of second-generation antipsychotic therapy (details not stated)	Psychotic relapse	Re-emergence or a switch to severe negative symptoms after chronic SCRA use warrants reassessment of the antipsychotic dose/choice, stimulant avoidance, and targeted psychosocial rehabilitation.
**Simmons et al., 2011 [54]**	Case series	M = 3	**Case 1** = 25 **Case 2** = 21 **Case 3** = 19	Smoked	Agitation, amnesia, bizarre behavior, paranoia, unspecified delusions	Case 1: IV lorazepam (4 mg), 2 L of normal saline led to recovery. Haloperidol (5 mg) for post-extubation agitation. Case 3: Observation only.	Acute psychosis with remission	Treat agitation first with benzodiazepines. Secure airway if hypoventilating. Use haloperidol only once vital signs are stable. Most patients recover within 12–24 h.
**Skryabin et al., 2019 [55]**	Observational study	M = 60	MA = 23.6 ± 3.5	NA	Catatonia, anxiety, motor agitation, Kandinsky–Clerambault syndrome, delusions of influence, automatisms, telepathy, thought broadcasting and insertion, delusional ideas of interpretation, persecutory delusions, delusional ideas, cenesthopathic automatisms, tactile hallucinations, pseudo-hallucinations, acute verbal hallucinations with threatening monologues or dialogues	High-dose antipsychotics and prolonged inpatient/ICU care were frequently required.	Acute psychosis with remission	SCRA users in the referenced 60-patient cohort needed higher doses and longer hospitalizations than cannabis users.
**Skryabin et al., 2018 [56]**	Longitudinal, observational cohort study	M = 46	MA = 23.2 ± 3.5	NA	Psychomotor agitation, anxious–depressive symptoms, mild hypomania, negative symptoms of schizophrenia, Kandinsky–Clerambault syndrome, persecutory delusions, paranoia, auditory and visual hallucinations	Neuroleptics were introduced on day 1 with detox measures. Choice (haloperidol vs. tiapride) was tailored to clinical variant. Benzodiazepines were used for psychomotor agitation (doses not specified).	Acute psychosis with remission/onset with persistence/psychotic relapse	Begin antipsychotic treatment immediately in SCRA-related psychosis, matching the drug class with the delirious/oneiroid/amentive pattern and autonomic burden. Integrate close follow-up because ≈17% of patients later show schizophrenic-process manifestation, making long-term psychiatric supervision essential.
**Sönmez et al., 2016 [57]**	Case report	M = 1	31	Smoked	Agitation, distressed mood, insomnia, ideation related to harming self and others, irritation, bursts of anger, delusions of persecution and reference, shape and content of thought altered	Inpatient olanzapine (20 mg/day × 10 days) led to complete resolution. The patient was discharged on the same dose and received cognitive-behavioral psychotherapy.	Acute psychosis with remission	Admit SCRA psychosis early, administer an adequate dose of a second-generation antipsychotic (olanzapine worked within a week), and schedule structured CBT to consolidate abstinence and reality testing.
**Sweet et al., 2017 [58]**	Case report	M = 1	47	Smoked	Psychomotor agitation, paranoia	Olanzapine ODT (10 mg) was ineffective. IM haloperidol (10 mg) + lorazepam (2 mg) + diphenhydramine (50 mg) achieved rapid control.	Acute psychosis with remission	In ED/acute-ward settings, treat SCRA-related agitation the same day: give an atypical IM antipsychotic (or haloperidol + lorazepam if unavailable), repeat q 30–60 min until calm, correct electrolytes, and watch for at least 6 h (symptoms may last up to 7 h).
**Tung et al., 2012 [59]**	Case report	M = 1	36	Smoked	Agitation, insomnia, dysphoric mood, persecutory delusion, disorganized thoughts and behavior, irrelevant speech, bizarre behavior, auditory hallucination	IM midazolam for rapid tranquillization + physical restraints on arrival. No antipsychotic started. Full resolution after 3 days of drug-free observation.	Acute psychosis with remission	A single benzodiazepine dose may suffice; if symptoms settle, avoid unnecessary antipsychotics and focus on substance-use assessment and education.
**Udow et al., 2018 [60]**	Case report	F = 1	70	Oral	Anxiety, persecutory delusions, bizarre visual hallucinations	Stopped nabilone and tapered pramipexole. Short trial of quetiapine (12.5 mg hs) (insufficient). Initiated clozapine (12.5 → 50 mg hs).	Acute psychosis with remission/onset with persistence	Older PD patients are highly vulnerable to SCRA-induced psychosis. First withdraw the offending drug and rationalize dopaminergic therapy. Use very-low-dose clozapine (with fludrocortisone or midodrine if needed) rather than dopamine-blocking antipsychotics.

Abbreviations: BDZ = benzodiazepines; BP = blood pressure; CBT = cognitive behavioral therapy; ED = emergency department; EPSs = extrapyramidal symptoms; FGA = first-generation antipsychotic; F = females; M = males; N = total subjects; HS = at bedtime; ICU = intensive care unit; IM = intramuscular; IV = intravenous; IVFs = intravenous fluids; LAI = long-acting injectable; MA = mean age; NA = not available; NEWS = National Early Warning Score; ODT = orally disintegrating tablet; PD = Parkinson’s disease; PRN = as needed; q6 h = every 6 h; QT = QT interval; SCRAs = synthetic cannabinoid receptor agonists; SGA = second-generation antipsychotic; TDS/TID = three times a day.

**Table 2:** Pharmacological management.

Therapeutic Domain	Evidence Base	Typical Dose Range Reported	Key Findings
**Benzodiazepines (BZDs)**	27/35 manuscripts, >700 pts	IV/IM lorazepam (2–6 mg), diazepam (10 mg TID), midazolam (1 mg)	Universal first-line agent for agitation, convulsions, or catatonia. As a monotherapy, it achieved full clinical resolution of mild-to-moderate intoxications within 6–24 h.
**Typical antipsychotics**	10/35 manuscripts (mainly from Eastern Europe)	Haloperidol (5–30 mg/day), IM chlorpromazine	Effective for florid psychosis but required high doses and close QT/EP symptom monitoring.
**Second-generation antipsychotics (SGAs)**	22/35 manuscripts	Olanzapine (10–20 mg/day), risperidone (2–6 mg/day), aripiprazole (10–20 mg/day)	Favored in Western cohorts; usually started after BZD. Time to remission: 24–72 h. Adherence problems prompted two reports of LAI paliperidone.
**Clozapine**	3 resistant cases	50–150 mg/day (adult), 12.5–50 mg/day (older PD patient)	Robust improvement where ≥2 other antipsychotics failed. Effective at lower doses than in primary schizophrenia.
**Anesthetic agents**	2 case series/reports	Propofol bolus/infusion	Enabled surgical airway or globe-repair procedures after extreme agitation or self-injury.
**Detox/supportive care**	Pediatric and ED cohorts	IV crystalloids, oxygen, B vitamins	In total, 70% of 1898 ED attendees required no psychotropics once hydrated and observed in a low-stimulus setting.

**Abbreviations:** BZDs = benzodiazepines; IV = intravenous; IM = intramuscular; TID = three times daily; SGAs = second-generation antipsychotics; LAI = long-acting injectable; PD = Parkinson’s disease; ED = emergency department; QT = QT interval; EP = extrapyramidal.

## Data Availability

Not applicable.

## Appendix A. Articles Excluded Based on Full Text

**Name, Year**	**Title**	**Motivation**
**Altintop, 2020**	A 4-Year Retrospective Analysis of Patients Presenting at the Emergency Department with Synthetic Cannabinoid Intoxication in Turkey	No treatment for SCRA-induced psychosis
**Altintop et al., 2019**	Assessment of Patients Admitted to Emergency Rooms with Synthetic Cannabinoid Intoxication: A Prospective Study	No treatment for SCRA-induced psychosis
**Benford et al., 2011**	Psychiatric Sequelae of Spice, K2, and Synthetic Cannabinoid Receptor Agonists	Data not available
**Gilley et al., 2021**	Synthetic Cannabinoid Exposure in Adolescents Presenting for Emergency Care	Does not deal with SCRA-induced psychosis
**O’Mahony et al., 2024**	HHC-induced psychosis: a case series of psychotic illness triggered by a widely available semisynthetic cannabinoid.	No treatment for SCRA-induced psychosis
**Ricci et al., 2023**	First episode psychosis with and without the use of cannabis and synthetic cannabinoids: Psychopathology, global functioning and suicidal ideation and antipsychotic effectiveness	No treatment for SCRA-induced psychosis
**van der Veer et al., 2011**	Persistent psychosis following the use of Spice	Data not available

## Appendix B. Specific SCRAs Identified

**Name, Year**	**Substance**
**Abouchedid et al., 2016 [26]**	JWH-18
**Altintas et al., 2016 [27]**	Unspecified SCRAs
**Barceló et al., 2017 [28]**	5F-ADB, MMB-2201
**Bassir et al., 2016 [29]**	Unspecified SCRAs
**Bebarta et al., 2012 [30]**	Spice
**Berry-Cabàn et al., 2013 [31]**	Spice
**Besli et al., 2015 [32]**	Unspecified SCRAs
**Bonaccorso et al., 2018 [33]**	Unspecified SCRAs
**Celofiga et al., 2014 [34]**	Unspecified SCRAs
**Di Petta et al., 2016 [35]**	Unspecified SCRAs
**Durand et al., 2015 [36]**	Unspecified SCRAs
**Every-Palmer et al., 2011 [37]**	JWH-018
**El Zahran et al., 2019 [38]**	Cumyl-4-cyano-BINACA
**Glue et al., 2013 [39]**	K2
**Haro et al., 2014 [40]**	JWH-081, JWH-250, JWH-203, JWH-019
**Helge Müller et al., 2009 [41]**	Spice
**Hermanns-Clausen et al., 2017 [42]**	AB-CHMINACA, MDMB-CHMICA
**Hoyte et al., 2012 [43]**	Unspecified SCRAs
**Hurst et al., 2011 [44]**	Spice
**Kekelidze et al., 2019 [45]**	JWH, AB-PINACA, TMCP
**Malik et al., 2021 [46]**	K2
**Monte et al., 2017 [47]**	Unspecified SCRAs
**Oluwabusi et al., 2012 [48]**	K2
**Ozer et al., 2016 [49]**	Unspecified SCRAs
**Peglow et al., 2012 [50]**	Spice
**Rahmani et al., 2013 [51]**	Unspecified SCRAs
**Roberto et al., 2016 [52]**	Unspecified SCRAs
**Satodiya et al., 2020 [53]**	K2
**Simmons et al., 2011 [54]**	JWH-018, JWH-073
**Skryabin et al., 2019 [55]**	Unspecified SCRAs
**Skryabin et al., 2018 [56]**	Spice
**Sönmez et al., 2016 [57]**	Unspecified SCRAs
**Sweet et al., 2017 [58]**	Unspecified SCRAs
**Tung et al., 2012 [59]**	Spice
**Udow et al., 2018 [60]**	Unspecified SCRAs

## Appendix C. Psychiatric Comorbidity

**Name, Year**	**Psychiatric Comorbidity**
**Abouchedid et al., 2016 [26]**	Depression
**Altintas et al., 2016 [27]**	NA
**Barceló et al., 2017 [28]**	No
**Bassir et al., 2016 [29]**	Schizophrenia, schizoaffective disorder, bipolar disorder, MDD, others
**Bebarta et al., 2012 [30]**	No
**Berry-Cabàn et al., 2013 [31]**	No
**Besli et al., 2015 [32]**	NA
**Bonaccorso et al., 2018 [33]**	Paranoid schizophrenia, schizoaffective disorder, bipolar disorder
**Celofiga et al., 2014 [34]**	Paranoid schizophrenia, undifferentiated schizophrenia
**Di Petta et al., 2016 [35]**	Previous psychosis related to substance abuse
**Durand et al., 2015 [36]**	No
**Every-Palmer et al., 2011 [37]**	Schizophrenia, schizoaffective disorder, borderline personality
**El Zahran et al., 2019 [38]**	No
**Glue et al., 2013 [39]**	Affective disorder, psychotic episodes
**Haro et al., 2014 [40]**	NA
**Helge Müller et al., 2009 [41]**	History of recurrent cannabis-induced psychotic episodes
**Hermanns-Clausen et al., 2017 [42]**	NA
**Hoyte et al., 2012 [43]**	NA
**Hurst et al., 2011 [44]**	No
**Kekelidze et al., 2019 [45]**	NA
**Malik et al., 2021 [46]**	Schizophrenia, SUD
**Monte et al., 2017 [47]**	NA
**Oluwabusi et al., 2012 [48]**	No
**Ozer et al., 2016 [49]**	No
**Peglow et al., 2012 [50]**	Post-traumatic stress disorder, SUD
**Rahmani et al., 2013 [51]**	No
**Roberto et al., 2016 [52]**	No
**Satodiya et al., 2020 [53]**	Schizophrenia
**Simmons et al., 2011 [54]**	No
**Skryabin et al., 2019 [55]**	SCRA dependence, cannabis use disorder, SCRA abuse
**Skryabin et al., 2018 [56]**	NA
**Sönmez et al., 2016 [57]**	No
**Sweet et al., 2017 [58]**	NA
**Tung et al., 2012 [59]**	No
**Udow et al., 2018 [60]**	Occasional visual hallucinations for many years with preserved insight

## Appendix D. Poly-Abuse

**Name, Year**	**Poly-Abuse (Substance)**
**Abouchedid et al., 2016 [26]**	LSD
**Altintas et al., 2016 [27]**	Cannabis, alcohol, stimulant, opioid
**Barceló et al., 2017 [28]**	Cannabis
**Bassir et al., 2016 [29]**	Cannabis
**Bebarta et al., 2012 [30]**	Acetaminophen, dextromethorphan, doxylamine
**Berry-Cabàn et al., 2013 [31]**	Mephedrone, cannabis, alcohol
**Besli et al., 2015 [32]**	Alcohol, amphetamines
**Bonaccorso et al., 2018 [33]**	Crack, cocaine, heroin, alcohol, MDMA, cannabis, legal highs polysubstance misuse
**Celofiga et al., 2014 [34]**	NA
**Di Petta et al., 2016 [35]**	Salvia divinorum, cocaine, efedrine, ketamine, alcohol
**Durand et al., 2015 [36]**	Cannabis
**Every-Palmer et al., 2011 [37]**	No
**El Zahran et al., 2019 [38]**	No
**Glue et al., 2013 [39]**	NA
**Haro et al., 2014 [40]**	NA
**Helge Müller et al., 2009 [41]**	No
**Hermanns-Clausen et al., 2017 [42]**	Amphetamines
**Hoyte et al., 2012 [43]**	No
**Hurst et al., 2011 [44]**	Cannabis, alcohol
**Kekelidze et al., 2019 [45]**	NA
**Malik et al., 2021 [46]**	NA
**Monte et al., 2017 [47]**	NA
**Oluwabusi et al., 2012 [48]**	NA
**Ozer et al., 2016 [49]**	No
**Peglow et al., 2012 [50]**	No
**Rahmani et al., 2013 [51]**	Cannabis, LSD, psilocybin, mushrooms, Spice, bath salts, oxycodone
**Roberto et al., 2016 [52]**	No
**Satodiya et al., 2020 [53]**	NA
**Simmons et al., 2011 [54]**	No
**Skryabin et al., 2019 [55]**	NA
**Skryabin et al., 2018 [56]**	NA
**Sönmez et al., 2016 [57]**	No
**Sweet et al., 2017 [58]**	NA
**Tung et al., 2012 [59]**	Polysubstance abuse
**Udow et al., 2018 [60]**	No

## Appendix E. RoBINS-I Assessment

**Author (Year)**	**Study Type**	**Confounding**	**Selection of Participants**	**Classification of Interventions**	**Deviations from Intended Interventions**	**Missing Data**	**Measurement of Outcomes**	**Selection of Reported Result**	**Overall Judgment**
Altintas et al. (2016) [27]	Single-center cross-sectional analysis	Serious—Confounding by psychiatric diagnosis and polysubstance use	Moderate—Participants were psychiatric patients, which limits representativeness	Low—Exposure classification via structured interview	Low—No deviations relevant	Moderate—Some missing records	Serious—Outcomes not blinded, limited validity	Moderate—Some selective emphasis	Serious risk of bias
Bassir et al. (2016) [29]	Retrospective review	Serious—Multiple confounders, no adjustment	Moderate—Hospitalized psychiatric patients only	Low—Exposure classification based on records	Low—No deviations relevant	Moderate—Some missing records	Serious—Outcomes not systematically validated	Moderate—Some selective emphasis	Serious risk of bias
Every-Palmer (2011) [37]	Cohort study	Serious—Multiple unmeasured confounders	Serious—Small forensic psychiatric sample, very selective	Low—Exposure classification consistent (self-report of JWH-018)	Moderate—No intervention deviations applicable	Moderate—Incomplete data from interviews	Serious—Outcomes subjective, not standardized	Serious—Results selectively described	Serious risk of bias
Glue et al. (2013) [39]	Retrospective observational study	Serious—Confounding by severity of illness	Moderate—All hospitalizations reviewed, small sample	Low—Exposure classification clear (SC identified)	Low—No deviations relevant	Moderate—Some missing documentation	Serious—Outcomes variable, not standardized	Moderate—Some selective reporting	Serious risk of bias
Hermanns-Clausen et al. (2018) [42]	Prospective observational study	Serious—Multiple confounding variables not controlled	Low—Consecutive ED patients included	Low—Exposure confirmed analytically	Low—No deviations, observational design	Moderate—Some missing clinical and lab data	Serious—Heterogeneous outcome measures across sites	Moderate—Selective reporting possible	Serious risk of bias
Hoyte et al. (2012) [43]	Observational study	Serious—Confounding by reporting patterns	Low—All NPDS cases included	Low—Exposure classification robust for poison center data	Low—Observational, no deviations relevant	Moderate—Some missing data in registry	Serious—Outcomes inconsistently categorized	Moderate—Registry limitations	Serious risk of bias
Kekelidze et al. (2019) [45]	Interventional study	Serious—No confounder adjustment	Moderate—Psychiatric hospital patients, limited representativeness	Low—Exposure classification by clinical record	Low—Observational, no deviations relevant	Moderate—Some incomplete records	Serious—Outcomes not validated	Moderate—Selective reporting likely	Serious risk of bias
Monte et al. (2017) [47]	Cohort study	Serious—Confounding by indication and severity, no comparator	Low—All cases from registry included consecutively	Low—Exposure classification robust (clinical registry)	Low—No deviations relevant, observational design	Moderate—Some incomplete clinical details	Serious—Outcomes heterogeneous across centers	Moderate—Registry structure limits selective reporting	Serious risk of bias
Skryabin et al. (2019) [55]	Observational study	Serious—No adjustment for confounding variables	Moderate—Participants were consecutive SC users but not population-based	Low—Exposure classification clear (self-report + clinical diagnosis)	Moderate—Deviations possible, treatment not standardized	Moderate—Some missing follow-up data	Serious—Outcomes not systematically validated	Moderate—Some selective reporting likely	Serious risk of bias
Skryabin et al. (2018) [56]	Longitudinal, observational cohort study	Serious—No confounder adjustment, polysubstance use not controlled	Moderate—Consecutive inpatients but not representative of general population	Low—Exposure classification clinical + confirmed SC use	Low—Observational, no intervention deviations	Moderate—Missing follow-up details for some patients	Serious—Psychiatric outcomes not systematically validated	Moderate—Potential selective emphasis in reporting	Serious risk of bias

## Appendix F. CARE Checklist—Case Reports

**Author (Year)**	**Title**	**Keywords**	**Abstract**	**Introduction**	**Patient Information**	**Clinical Findings**	**Timeline**	**Diagnostic Assessment**	**Therapeutic Intervention**	**Follow-Up and Outcomes**	**Discussion**	**Patient Perspective**	**Informed Consent**
Abouchedid et al. (2016) [26]	Present	Present	Present	Present	Present	Present	Absent	Present	Present	Present	Present	Absent	Present
Barceló et al. (2017) [28]	Present	Present	Absent	Present	Present	Present	Absent	Present	Present	Present	Present	Absent	Absent
Bebarta et al. (2012) [30]	Present	Present	Present	Present	Present	Present	Absent	Present	Present	Present	Present	Absent	Absent
Berry-Cabán et al. (2013) [31]	Present	Present	Present	Present	Present	Present	Absent	Present	Present	Present	Present	Absent	Absent
Besli et al. (2015) [32]	Present	Present	Present	Present	Present	Present	Absent	Present	Present	Present	Present	Absent	Absent
Bonaccorso et al. (2018) [33]	Present	Present	Present	Present	Present	Present	Absent	Present	Present	Present	Present	Absent	Absent
Celofiga et al. (2014) [34]	Present	Present	Present	Present	Present	Present	Absent	Present	Present	Present	Present	Absent	Absent
Di Petta (2016) [35]	Present	Absent	Absent	Present	Present	Present	Absent	Present	Present	Present	Present	Absent	Absent
Durand et al. (2015) [36]	Present	Present	Present	Present	Present	Present	Absent	Present	Present	Present	Present	Absent	Absent
El Zahran et al. (2018) [38]	Present	Present	Absent	Present	Present	Present	Absent	Present	Present	Present	Present	Absent	Absent
Haro et al. (2014) [40]	Present	Absent	Absent	Present	Present	Present	Absent	Present	Present	Present	Present	Absent	Absent
Hurst et al. (2011) [44]	Present	Present	Absent	Present	Present	Present	Absent	Present	Present	Present	Present	Absent	Absent
Malik et al. (2020) [46]	Present	Present	Present	Present	Present	Present	Absent	Present	Present	Present	Present	Absent	Absent
Oluwabusi et al. (2012) [48]	Present	Absent	Absent	Present	Present	Present	Absent	Present	Present	Present	Present	Absent	Absent
Ozer et al. (2016) [49]	Present	Present	Present	Present	Present	Present	Absent	Present	Present	Present	Present	Absent	Present
Peglow et al. (2012) [50]	Present	Present	Present	Present	Present	Present	Absent	Present	Present	Present	Present	Absent	Absent
Rahmani et al. (2014) [51]	Present	Present	Present	Present	Present	Present	Absent	Present	Present	Present	Present	Absent	Absent
Roberto et al. (2016) [52]	Present	Present	Present	Present	Present	Present	Absent	Present	Present	Present	Present	Absent	Present
Satodiya & Palekar (2020) [53]	Present	Present	Present	Present	Present	Present	Absent	Present	Present	Absent	Present	Absent	Present
Sönmez & Köşger (2016) [57]	Present	Present	Present	Present	Present	Present	Absent	Present	Present	Present	Present	Absent	Absent
Sweet et al. (2017) [58]	Present	Present	Present	Present	Present	Present	Absent	Present	Present	Present	Present	Absent	Absent
Tung et al. (2012) [59]	Present	Present	Present	Present	Present	Present	Absent	Present	Present	Present	Present	Absent	Absent
Udow et al. (2018) [60]	Present	Present	Present	Present	Present	Present	Absent	Present	Present	Present	Present	Absent	Absent
